# Effects of sanitation and hygiene perceptions on international travelers' health, travel plans and trip experiences in India

**DOI:** 10.3389/fpubh.2022.1042880

**Published:** 2022-11-30

**Authors:** Rishita Chandra, Sakshi Supehia, Bhola Nath, Charu Chhetri, Ranjeeta Kumari, Kumari Damayanti Joshi, Roshan Sharma, Jatin Chaudary, Kishor Joshi, Ramesh Bhatta, Chhavi R. Bhatt

**Affiliations:** ^1^Department of Community and Family Medicine, AIIMS, Rishikesh, Uttarakhand, India; ^2^Department of Community Medicine, Dr RP Government Medical College, Kangra, Himachal Pradesh, India; ^3^Department of Community and Family Medicine, AIIMS Raebareli, Uttar Pradesh, India; ^4^Department of Community Medicine, Doon Medical College, Dehradun, Uttarakhand, India; ^5^School of Public Health and Preventive Medicine, Monash University, Melbourne, VIC, Australia; ^6^School of Education, Deakin University, Melbourne, VIC, Australia; ^7^Center for Urban Research, RMIT University, Melbourne, VIC, Australia; ^8^Humsafar Trust, Mumbai, Maharashtra, India; ^9^School of Physiotherapy and Allied Health, Sardar Bhagwan Singh Post Graduate Institute of Biomedical Sciences and Research, Dehradun, India; ^10^Yeti Health Science Academy, Kathmandu, Nepal; ^11^Purbanchal University, Biratnagar, Nepal; ^12^School of Clinical Sciences at Monash Health, Monash University, Melbourne, VIC, Australia

**Keywords:** diarrhea, hygiene, sanitation, travel health, travel experience

## Abstract

**Background:**

International travelers often experience travelers' diarrhea. However, there is paucity of data on whether self-reported gastrointestinal symptoms influence travelers' perceptions of adequacy of sanitation and hygiene services encountered during travel, and to what degree their travel plans, and overall trip experience are impacted.

**Methods:**

A cross-sectional face-to-face survey was conducted amongst international travelers in India. Data collected included socio-demographics, travel characteristics, self-reported occurrence and frequency/severity of gastrointestinal symptoms, perceptions of sanitation and hygiene encountered, and adverse effects of symptoms on travel plans and trip experiences. Chi-square tests and logistic regression were performed to describe differences and associations between categorical variables.

**Results:**

Of the 300 international travelers surveyed, 46.3% experienced diarrhea. At least two thirds of travelers perceived the quality of sanitation (67.0%) and hygiene (70.0%) encountered to be inadequate. Perceptions of inadequate sanitation (adjusted OR = 3.0; 95% CI 1.7–5.5) and poor hygiene (adjusted OR = 7.7; 95% CI 4.1–15.5) were higher among travelers who experienced diarrhea. Additionally, both higher likelihood of travel plans being affected (adjusted OR = 10.7; 95% CI 5.1–23.6) and adverse impacts on overall trip experience (adjusted OR = 2.8; 95% CI 1.4–5.8) were reported among those who experienced diarrhea.

**Conclusions:**

More than two thirds of travelers surveyed in India experienced inadequate sanitation and hygiene services, with perceptions influenced by occurrence and frequency of diarrhea. Self-reported diarrhea was also associated with adverse effects on travel plans and overall trip experience. While these results may seem intuitive, they have important implications and suggest that improving sanitation and hygiene standards in India could potentially enhance tourism.

## Background

Inadequate sanitation and hygiene pose a major health challenge in many low- and middle-income countries, including India. In a 2018 Indian Government report, on average the proportions of households with access to an improved water supply or improved sanitation facilities (latrines) were 96%; ~85% (of both rural and urban populations accessing latrine) respectively. Similarly, the proportions of households practicing hand washing with water and soap/detergent after defecation and before meals were 74 and 35.8%, respectively (1). Access to an improved water supply and latrines does not necessarily equate with optimal sanitation management practices, which ideally should include safe disposal of septic waste to avoid environmental contamination. Furthermore, the majority of public toilets in India lack proper infrastructure, cleanliness, adequate water supply and hand washing facilities, therefore failing to meet optimal hygiene standards (2, 3).

Travelers who visit countries with poor hygiene and sanitation are at increased risk of exposure to diseases transmitted through the feco-oral route (4). Consumption of fecally contaminated water or food, and exposure to fecally contaminated environments, are associated with a range of diseases affecting travelers (5), in particular travelers' diarrhea (TD). TD is the most frequent illness reported amongst international travelers to developing nations (6), reported in up to half (7).

Travelers visiting India are at higher risk of acquiring travel-associated gastrointestinal illnesses than travelers to other regions (8–11). A study reporting a case of diarrhea in a traveler returning from India emphasized on the duration of symptoms as a significant factor for identifying the cause of diarrhea among travelers (12). The evidence on traveler's diarrhea suggests its association with the presence or predominance of bacteria such as Carbapenemase-producing Enterobacterales in tropical regions like India, which happens to be a reservoir for multi-drug resistant bacteria (13–15).

India, a popular tourist destination with about 10.6 million international tourists visiting in 2018 (16), is predicted to become the third-largest tourism economy worldwide by 2028 (17). However, the fact that perceived health risk is strongly associated with the tourists' decision and travel behavior, is well-established. Tourism industry is significantly impacted by such consumer behavior that includes change in destination, postponement of trips etc. (18). Negative experiences may impact future travel plans and tourists avoid destinations with more perceived health risks (19). Poor sanitation and hygiene are recognized as factors that negatively impact tourism services (20–22). Previous studies have demonstrated a strong correlation between improving hygiene standards at a travel destination and declining rates of diseases transmitted *via* the fecal-oral route among travelers (5). A study by Kozak et al. reported that tourist's travel experience is inversely proportional to the perceived risk (6). Therefore, overall improvement in sanitation and hygiene standards in India could potentially decrease the rate of fecal-orally transmitted diseases amongst visiting travelers and their perceptions of future travel would not be negatively influenced.

Despite the reportedly high rates of fecal-orally transmitted diseases in travelers (5–7), it is currently unknown whether gastrointestinal illness and perceptions of sanitation and hygiene conditions at a destination have an impact on trip experiences and future travel plans amongst tourists.

The purpose of this study was to: (i) describe associations between occurrence (including frequency and severity) of self-reported gastrointestinal illness and perceptions of inadequate sanitation/hygiene among tourists in India, and (ii) determine whether negative health experiences (e.g., diarrhea) and/or negative perceptions of sanitation or hygiene influence travel plans or travel experiences.

## Methods

### Study design

This was a cross-sectional study conducted amongst international travelers visiting the Northern Indian city of Rishikesh, located 250 km northeast of the capital city of India, New Delhi. An international traveler was defined as any tourist with a nationality other than Indian who had been in India for at least 7 days. A non-probability convenience sampling approach was used to recruit 300 travelers for the study.

The sample size was calculated on the likely prevalence of self-reported gastrointestinal illnesses (i.e., travelers' diarrhea). Since this was a pilot, exploratory study with a novel outcome, we made the following assumptions for assessing an association between prevalence of diarrhea in past 7 days and perception of sanitation experience among international travelers to India: (i) 34% of international travelers to India experience diarrhea, (ii) 2/3rd (67%) the travelers who experience diarrhea and half (50%) of the travelers who do not experience diarrhea report their perception of sanitation experience in India to be negative (inadequate), (iii) this results in the calculated odds ratio (effect size) of 2.03, and (iv) 5% precision (two-tailed), and 80% power. These assumptions therefore resulted in the total sample size of 300.

De-identified data were collected between September 2019 and February 2020 through face-to-face surveys using a paper-based questionnaire with closed-ended items. The survey tool design (Appendix in Supplementary material) was based on data collection tools available in travel medicine and public health literature (3, 10, 11). Local travel, tour, and restaurant/hotel operators were contacted and asked for permission to approach their guests for the study. Where permission was granted, trained research assistants approached guests of these operators, as well as tourists visiting local tourist attractions. An explanatory statement was provided to all those approached and written informed consent was obtained from those who agreed to participate in the study.

### Ethics approval

The study protocol was reviewed and approved by the human research ethical committees of Governmental Doon Medical College in India (IEC-049, GDMC) and Monash University in Australia (MUHREC 20359).

### Participants and data collection

Eligible participants were international travelers aged 18 years or older who had already been in India for at least 7 days. Data collected included information on socio-demographics, travel characteristics (e.g., purpose and duration of the visit), food and drink habits, hand hygiene practices, and medication use. Additionally, data on self-reported health outcomes (including occurrence and frequency of gastrointestinal symptoms such as diarrhea, nausea/vomiting, loss of appetite and abdominal pain) and their relationship to travel plans/experiences, medical advice or vaccinations prior to the trip, and access to and perceptions of sanitation and hygiene services were also collected.

Variables of interest included perception of sanitation and/or hygiene as inadequate, occurrence of self-reported diarrhea in the past 7 days (23, 24), self-reported severity of diarrhea (mild, moderate, or severe) (25); frequency of diarrhea (always, often, sometimes, rarely), occurrence of other gastrointestinal symptoms (nausea/vomiting, loss of appetite, abdominal pain, including cramping/stomach ache, bloody stool) (9–11), alteration in current and future travel plans and adverse impact on trip experience.

### Data analysis

De-identified data from paper-based surveys were entered and stored in a customized REDCap^TM^ database (HELIX, Monash University) by research assistances in India. Raw data were extracted for analysis.

Frequencies and proportions were computed for descriptive results. Chi-square tests were performed to evaluate statistical differences among categorical data. Logistic regression was performed to assess associations between (i) occurrence and/or severity of self-reported gastrointestinal illnesses and perceptions of sanitation and hygiene experiences among travelers, and (ii) negative health experiences (e.g., diarrhea) and/or negative perceptions of water, sanitation and hygiene facilities and travelers' future travel planning.

Frequency of diarrhea and other gastrointestinal illness symptoms were grouped into less frequent (sometimes and rarely) and more frequent (always and often) categories. Similarly, duration of stay in India was also grouped into “ <2 weeks” and “equal or more than 2 weeks.” Association of the duration of time spent in India with the occurrence and frequency of diarrhea and other gastro-intestinal symptoms was estimated using the chi-square test. Bivariate logistic regression was used to estimate odds ratios with 95% CIs after adjusting for demographic factors. For all statistical tests, *p* < 0.05 were considered statistically significant. All statistical analyses were carried out using STATA (version 14, StataCorp, College Station, TX, USA) and SPSS version 23.

## Results

### Demographic and travel characteristics

**Figure 1:**
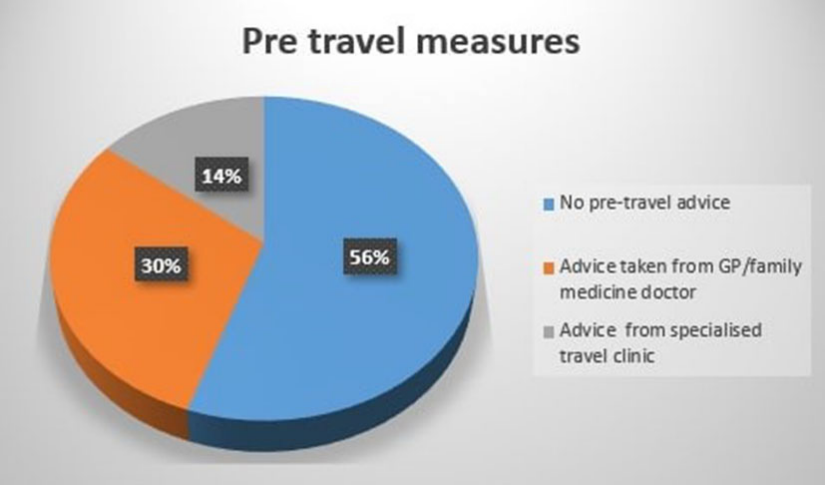
Pre-travel medical advice taken by travelers.

**Figure 2:**
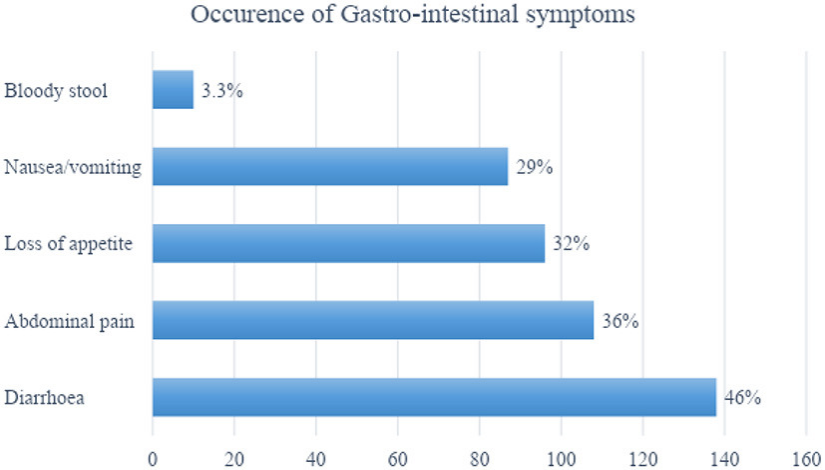
Occurrence of gastro-intestinal symptoms among travelers.

As illustrated in Table 1, majority of the travelers were females being 61% (*N* = 300). Most of the travelers were of 40 years or less. The traveler's were from across the globe with 69% from China, the USA, the UK, Australia, Italy, Canada, France, Russia, and Germany. Majority of the tourists were on vacation and about 17% visited India for education or volunteer work. Approximately half of the travelers' planned their trip for more than 4 weeks with more than 40% who preferred to stay in a hotel. Antibiotics were the most common medicine carried by about 60% of the travelers and 70% preferred bottled water. About half of the travelers didn't seek any medical advice prior to their trip as illustrated in Figure 1. The incidence of diarrhea were reported by 46% of the travelers (Figure 2).

**Table 1:** Travelers' demographic and travel characteristics.

**S. no**.	**Variables (*n* = 300)**	**Frequency (%)**
**1**	**Sex**	
	Females	182 (60.7%)
	Males	117 (39.0%)
	No answer	1 (0.3%)
**2**	**Age (years)**	
	18–30	151 (50.3%)
	31–40	115 (38.4%)
	>40	34 (11.3%)
**3**	**Country of origin**	
	China	42 (14.0%)
	USA	35 (11.7%)
	UK	27 (9.4%)
	Australia	23 (7.7%)
	Italy	22 (7.3%)
	Canada	17 (5.7%)
	France	16 (5.3%)
	Russia	15 (5.0%)
	Germany	10 (3.3%)
	Other countries	93 (30.6%)
**4**	**Ethnicity**	
	White	212 (70.7%)
	Asian (Far-East)	33 (11.0%)
	Asian (Indian subcontinent)	16 (5.3%)
	Hispanic/Latino	14 (4.7%)
	Black	10 (3.3%)
	Bi/Multi-racial	10 (3.3%)
	Middle Eastern	5 (1.7%)
**5**	**Highest level of education**	
	University degree	188 (62.7%)
	Secondary school	100 (33.3%)
	Primary school	12 (4.0%)
**6**	**Travel reasons**	
	Tourism/vacation	179 (59.7%)
	Educational/research	40 (13.3%)
	Volunteer/missionary	14 (4.7%)
	Visiting friends/relatives	12 (4.0%)
	Business	8 (2.7%)
	Other	47 (15.7%).
**7**	**Planned travel duration**	
	>4 weeks	148 (49.3%)
	3–4 weeks	92 (30.7%)
	1–2 weeks	51 (17.0%)
	<1 week	9 (3.0%)
**8**	Traveling partners^*^	
	With friend(s)/family	162 (54.4%)
	None	136 (45.6%)
**9**	Time already spent in India^*^	
	<2 weeks	112 (37.5)%
	2–4 weeks	111 (37.1)%
	>4 weeks	76 (25.4)%
**10**	**Stay venues**	
	Hotel	125 (41.7%)
	Guesthouse	57 (19.0%)
	Home stay/friend's home	26 (8.7%)
	Apartment	14 (4.7%)
	Other (non-specified)	78 (26.0%)
**11**	**Underlying health conditions**	
	No	275 (91.7%)
	Yes	25 (8.3%)
**12**	**Hepatitis A vaccination**	
	Never received	155 (51.7%)
	Single dose received (>12 months ago)	29 (9.7%)
	Two doses received (completed course)	58 (19.3%)
	Received within 12 months	58 (19.3%)
**13**	Typhoid vaccination within last 3 years^*^	
	No	200 (66.9%)
	Yes	99 (33.1%)
**14**	**Travelers who carried medicines/drugs**	
	Probiotics	79 (26.3%)
	Anti-biotics	180 (60%)
	Anti-emetics	77 (25.7%)
	Anti-motility	44 (14.7%)
**15**	**Travelers' intake of drinking water** ^†^	
	Bottled water	210 (70%)
	Boiled water	90 (30%)
	Tap water	19 (6.3%)
	UV treated water	46 (15.3%)
	Chlorinated water	21 (7%)

* Data missing in 1 (Time already Spent in India and Typhoid vaccination status) or 2 (Traveling partners) patients.

† Multiple choice question.

### Perception of sanitation and hygiene experiences

Perceptions of inadequate sanitation and inadequate hygiene were reported by about 67 and 70% of travelers respectively. These perceptions did not differ across sexes (*p* > 0.05). One in four travelers (24.1%) reported difficulties in finding a public toilet; just over half reported they were able to find a public toilet when needed. Almost half the travelers (43.8%) reported that most public toilets did not have a hand washing basin with soap or hand sanitizer. Approximately equal proportions of travelers reported that their experiences of toilet and hand-washing facilities were better than (28%) or worse than (28.7%) expected. The perception of inadequate sanitation and hygiene was found to be different according to the use of water (e.g., bottled water, boiled water and tap water). The perception of sanitation was significantly different among the travelers using bottled water and boiled water (Chi^2^ = 4.95, *p* = 0.03; Chi^2^ = 9.17, *p* = 0.002, respectively). Similarly, the perception of inadequate hygiene was found to be significantly different among the travelers using tap water (Chi^2^ = 4.05, 5.24 for bottled and boiled water respectively). Supplementary Tables A,B summarize travelers' perception of sanitation and hygiene experiences, and frequency of gastro-intestinal symptoms.

### Travel-related gastro-intestinal symptoms, frequency/severity, and consequences

Health outcome data for travelers is reported in Table 1. Forty six percent of travelers experienced diarrhea in the past 7 days; most (88%) had mild (tolerable) symptoms. Other gastro-intestinal symptoms experienced included abdominal pain (36%), loss of appetite (32%) and nausea/vomiting (29%). Of the travelers who experienced gastro-intestinal symptoms during their trip, nearly one-third (32.9%) reported that their symptoms adversely affected their overall trip experience. Over one-third of travelers reported that their gastro-intestinal symptoms affected their travel plans, of whom 51% stopped traveling, 26% needed to change travel plans, 15% needed a medical consultation, and 8% needed hospitalization.

### Associations between gastro-intestinal symptoms, perception of inadequate sanitation or inadequate hygiene and impact on travel plans

Travelers who reported symptoms of diarrhea in the past 7 days were significantly more likely to report perceptions of inadequate sanitation (adjusted OR = 3.0; 95% CI 1.7–5.5, *p* = 0.0001) or inadequate hygiene (adjusted OR = 7.7; 95% CI 4.1–15.5, *p* < 0.0001) compared to those without diarrhea (Table 2). These associations were even more pronounced among travelers who reported frequent diarrheal symptoms (inadequate sanitation: adjusted OR = 5.5; 95% CI 1.3–3.1, *p* = 0.03; inadequate hygiene: adjusted OR = 38.4; 95% CI 3.0–2,090.4, *p* = 0.03). Additionally, travelers who had frequent loss of appetite or abdominal pain were also significantly more likely to have a perception of inadequate sanitation or hygiene experiences compared with those who had less frequent symptoms.

**Table 2:** Associations between occurrence, frequency of gastro-intestinal symptoms, and perception of inadequate hygiene and inadequate sanitation experience.

**Gastro-intestinal symptoms** ^†^		**Perceived inadequate hygiene**						**Perceived inadequate sanitation**					
		**No**	**Yes**	**Crude OR (95% CI)**	***p* value**	Adjusted OR^*^(95% CI)	***p* value**	**No**	**Yes**	**Crude OR (95% CI)**	***p* value**	Adjusted OR^*^(95%CI)	***p* value**
Occurrence of diarrhea	No	72	88	Ref				70	90	Ref			
	Yes	17	121	5.8 (3.3–10.8)	0	7.7 (4.1–15.5)	<0.0001	29	109	2.9 (1.8–4.9)	0.00	3.04 (1.7–5.5)	0.0001
Frequency of diarrhea	Less frequent	16	86	Ref				25	77	Ref			
	More frequent	1	34	6.3 (1.2–116.4)	0.08	38.4 (3.0–2,090.4)	0.02	3	32	3.5 (1.1–15.3)	0.05	5.46 (1.3–3.1)	0.03
Loss of appetite	Less frequent	88	186	Ref				98	176	Ref			
	More frequent	1	25	11.8 (2.4–213.0)	0.01	12.2 (2.4–223.4)	0.01	1	25	13.9 (2.9–250.5)	0.01	12.84 (2.51–235.7)	0.01
Abdominal pain	Less frequent	86	178	Ref				96	168	Ref			
	More frequent	3	33	5.3 (1.8–22.5)	0.00	5.80 (1.9–25.8)	0.00	3	33	6.3 (2.2–26.6)	0.00	5.84 (1.9–25.8)	0.006

* Adjusted for age, sex, education, ethnicity, and underlying health conditions.

† Self-reported.

Among those with self-reported diarrhea in the past week, travel plans were more likely to have been impacted (adjusted OR = 10.6; 95% CI 5.1–23.7, *p* < 0.001) and the overall trip experience adversely affected (adjusted OR = 2.8; 95% CI 1.4–5.8 *p* = 0.004). Adverse impacts on both travel plans and on overall trip experience were also more likely to be reported by those who reported perceptions of inadequate sanitation (adjusted OR = 2.3; 95% CI 1.2–4.6, *p* = 0.01; and adjusted OR = 2.5; 95% CI 1.1–5.8, *p* = 0.02, respectively) or inadequate hygiene (adjusted OR = 3.1; 95% CI 1.5–6.6, *p* = 0.002; and OR = 3.6; 95% CI 1.6–9.2, *p* = 0.003, respectively).

### Associations between gastro-intestinal symptoms, and receipt of medical advice prior to the trip, purpose of travel, duration of travel and type of accommodation

Travelers who experienced diarrhea in the past 7 days were more likely to have spent more time in India and they were also significantly more likely to have sought medical advice prior to trip. In Table 3, the association between gastro-intestinal symptoms and above-mentioned variables are represented.

**Table 3:** Associations between gastro-intestinal symptoms and receipt of medical advice prior to the trip, purpose of travel, duration of travel and type of accommodation.

**Gastro-intestinal symptoms (*n* = 300)^†^**	**Receipt of medical advice prior to trip**			**Purpose of travel**			**Duration of travel/stay**			**Type of accommodation**		
	**Odd's ratio (95% CI)**	**Chi-square value**	*p* value^*^	**Odd's ratio (95% CI)**	**Chi-square value**	*p* value^*^	**Odd's ratio (95% CI)**	**Chi-square value**	*p* value^*^	**Odd's ratio (95% CI)**	**Chi-square value**	*p* value^*^
**Occurrence of diarrhea**												
No	Ref	14.53	0.00	Ref	3.88	0.04	Ref	7.56	0.00	Ref	7.72	0.00
Yes	0.4 (0.25–0.64)			1.59 (1.00–2.53)			1.93 (1.2–3.1)			1.93 (1.21–3.10)		
**Nausea and vomiting**												
Less frequent	Ref	3.58	0.00	Ref	0.19	0.66	Ref	1.47	0.23	Ref	1.02	0.31
More frequent	0.41 (0.15–1.08)			0.81 (0.32–2.03)			1.74 (0.7–4.3)			1.59 (0.64–3.95)		
**Loss of appetite**												
Less frequent	Ref	5.81	0.01	Ref	0.00	0.96	Ref	1.44	0.22	Ref	0.44	0.50
More frequent	0.36 (0.16–0.85)			1.01 (0.45–2.27)			0.94 (0.4–1.9)			0.75 (0.32–1.73)		
**Abdominal pain**												
Less frequent	Ref	12.56	0.00	Ref	0.80	0.30	Ref	0.26	0.87	Ref	1.06	0.30
More frequent	0.26 (0.12–0.57)			1.37 (0.68–2.77)			1.63 (0.7–3.6)			1.44 (0.71–2.9)		

† Detailed distribution is given in Supplementary Tables C (I–IV).

* Significance level <0.05.

### Impacts on future travel

Nearly all of the travelers (*n* = 296) reported that they would consider altering their future travel plans due to their sanitation and hygiene experiences by selecting better accommodation (45.9%, *n* = 136), avoiding rural areas (17.9%, *n* = 53), or making other itinerary changes (36.1%, *n* = 107). Further, 41.3% (*n* = 124) of travelers reported that their sanitation and hygiene experiences would influence where they choose to travel in the future. When considering their experiences of water, sanitation, and hygiene facilities in India, 32% of travelers either said they would not recommend (10.1%, *n* = 30) or were unsure about recommending (22%, *n* = 65) a visit to India.

## Discussion

This study assessed the associations between travelers' perceptions of sanitation and hygiene conditions during their time in India, the occurrence of diarrhea and gastrointestinal symptoms, and the impact of symptoms on travel plans and overall trip experiences.

Our results suggest that sanitation and hygiene conditions are inadequate for travelers in India, with nearly a quarter of the travelers reporting difficulty finding a public toilet when needed, more than one third reporting that most of the public toilets they used did not have a working flush, and almost 45% reporting a lack of hand washing basins with soap or hand sanitizer. This may be not surprising given the known inadequacies of sanitation and hygiene in India (1, 26) and its impact on diarrheal deaths among the endemic population, especially among children <5 years of age (25). Despite this, 28% of travelers mentioned that their experiences of toilet and hand washing facilities were better than they had expected. About 56% travelers carried an antibiotic and approximately 25% travelers carried anti-emetics and probiotics, with 15% carrying medicines for anti-motility. Among all travelers, 70% preferred bottled water for drinking.

In general, adequate sanitation and hygiene are important for a pleasurable travel experience and their absence may impact travelers' perceptions of a destination and may reduce tourist arrivals (16, 27, 28). The current study suggested that among those with self-reported diarrhea in the past week, travel plans were more likely to have been disrupted and the overall trip experience adversely affected as compared to those without diarrhea. In addition to the need for adequate sanitation and hygiene, the factors concerning overall travel-related health also include pre-travel measures. Approximately half of the travelers did not take any medical advice prior to the trip and the same proportion of travelers were neither vaccinated for Hepatitis A nor for typhoid. The most common drugs carried by the travelers were antibiotics. These findings of the current study align with the results of the study conducted by Bhajoni et al., suggesting a lack of promptness for pre-travel health measures for long-distance travels (29). It is reported in the literature that the decision of pre-travel vaccination is of utmost importance and depends on the assessment of risk an individual might be exposed (e.g., duration of stay, age of traveler, risk of activities they are planning during their trip etc.) (30). Hence, the study in alignment with the evidence reported in literature (31) suggests that there is a need to take pre-travel health measures sincerely and travelers should be aware enough in advance about the associated risks with international travels and improve the preventative health seeking behavior. The evidence in literature suggests that the safety of food has an influence on the destination choice of the travelers (32). This study highlighted significant association between GI symptoms and type of accommodation. However, adverse impacts on both travel plans and on overall trip experience were reported by those who had perceptions of poor sanitation and poor hygiene experiences.

Prior to the COVID-19 pandemic, the travel and tourism industry was one of the top export services for revenue generation, accounting for more than 10% of the World's Gross Domestic Product (33). This industry is vulnerable to people's perceptions, and if sanitation facilities are suboptimal or travelers are dissatisfied with their travel experience, they are less likely to visit again or recommend the destination to others. Therefore, improving sanitation and hygiene not only has obvious importance for the health of the local population but may also have an impact on the local tourism industry. This, in turn, helps in boosting the local and national economy mainly by generating earnings from foreign exchange and revenue (34). For India, it has been estimated that a reduction in the risk of travel-related infectious diseases including malaria, dengue and others would increase tourist arrivals by more than 8%. The implications for tourism could be considerable should the risks of hygiene and sanitation-related travel diseases (e.g., diarrhea and other gastrointestinal symptoms) are taken into account. This is particularly obvious in the era of post-COVID tourism where travelers' are expected to practice improved hygiene and sanitation (e.g., hand-washing) services.

Travelers who stay in home or other stay facilities with poor infrastructure, and those who engage in eco-tourism activities have been reported previously to be particularly susceptible to exposure to sub-optimal sanitation and hygiene conditions and practices (27, 28). In our study, nearly 61% of travelers stayed in hotels/ guesthouse, and these travelers had higher odds of perceiving hygiene to be inadequate.

Occurrence of diarrhea in travelers has a range of potential associated morbidities beyond the acute episode, including disruption of the gastrointestinal micro biome and an increased risk of colonization with multi-drug resistant organisms (35). This is particularly relevant for travelers to India given the high burden of multi-drug resistant infections among Indian populations (36). Evidence reports that ~50% of travelers from Asia carry an extended-spectrum beta-lactamase producing Enterobacteriaceae, and can result in a potential source for spread in their home countries (28). Occasionally, irritable bowel syndrome symptoms may occur among returnee travelers following the onset of traveler's diarrhea (37). A recent study conducted among Finish international travelers showed that travelers' diarrhea was a major risk factor for urinary tract infection, particularly among women (38).

We found that reported experiences of inadequate sanitation and hygiene conditions were higher among tourists who experienced diarrhea, particularly those with frequent symptoms. Gastrointestinal symptoms were also associated with travel plan disruptions, adverse impacts on the overall trip experience and potential impacts on future travel plans, such as consideration of better accommodation or changing itineraries. These results clearly indicate potential negative effects of inadequate sanitation and hygiene services on current and future international travelers.

This study has some limitations. The data were only collected from Rishikesh, the Yoga capital of the world, a popular international travel destination for spiritual and adventure tourism (39, 40). Additionally, it is possible that travelers who had experienced diarrhea or other gastrointestinal symptoms during the trip and consequently had stronger negative perceptions of inadequate sanitation and hygiene conditions may have been more likely to participate. Though food hygiene could also be associated with sanitation and hygiene perceptions, our study did not collect data on food hygiene related services or practices of the travelers. Also, the sample size, while adequate to find statistically significant results, was too small to enable generalizability of findings, and future research could explore this in other settings.

In conclusion, although it may seem intuitive that gastrointestinal illness during travel impacts on perceptions regarding the adequacy of sanitation and hygiene services, disruption to travel plans and overall trip satisfaction, to the best of our knowledge, this has not been explicitly studied previously. The poor perceptions of sanitation and hygiene practices together with their negative impact on overall trip experiences make the travel and tourism sector in India vulnerable. Therefore, it could be suggested that the Indian tourism sector should consider improving sanitation and hygiene issues in the country, which improves the health of both the local population as well as international travelers in the country. Also, in order to improve travel-related health, travelers should be sincere about their pre-travel measures and preventative health management. The precautionary measures on individual level to minimize gastrointestinal symptoms (e.g., diarrhea) should be encouraged. However, the need for maintaining sanitation and hygiene is definitely a state issue and importance of implementing such policies cannot be queried.

## Data availability statement

The raw data supporting the conclusions of this article will be made available by the authors, without undue reservation.

## Ethics statement

The studies involving human participants were reviewed and approved by the Human Research Ethical Committees of Governmental Doon Medical College in India (IEC-049, GDMC) and Monash University in Australia (MUHREC 20359). The patients/participants provided their written informed consent to participate in this study.

## Author contributions

RC: investigation, formal analysis, writing—original draft, and writing—review and editing. SS: investigation, data curation, formal analysis, and writing—review and editing. BN: methodology, data curation, writing—review and editing, resources and editing, funding acquisition, and supervision. CC: conceptualization, methodology, investigation, data curation, writing—review and editing. RK: investigation, data curation, writing—review and editing, and resources. KDJ: conceptualization, data curation, and project administration. RS: formal analysis and writing—review and editing. RB: conceptualization, methodology, and writing–review and editing. JC and KJ: investigation, data curation, and writing—review and editing. CB: conceptualization, methodology, investigation, data curation, writing—original draft, writing—review and editing, resources and supervision. All authors contributed to the article and approved the submitted version.

## Conflict of interest

The authors declare that the research was conducted in the absence of any commercial or financial relationships that could be construed as a potential conflict of interest.

## Publisher's note

All claims expressed in this article are solely those of the authors and do not necessarily represent those of their affiliated organizations, or those of the publisher, the editors and the reviewers. Any product that may be evaluated in this article, or claim that may be made by its manufacturer, is not guaranteed or endorsed by the publisher.
