# Supplementary material for: Effects of sanitation and hygiene perceptions on international travelers' health, travel plans and trip experiences in India
Source: Front Public Health. 2022 Nov 30;10:1042880. doi: 10.3389/fpubh.2022.1042880 (PMC9774491; doi:10.3389/fpubh.2022.1042880)
Supplement: Supplementary file 1 [file Table_1.DOCX]

**Supplementary tables**

**Table A: Travellers’ perception of sanitation, hygiene and symptoms**

| **Perception of sanitation, hygiene and symptoms** | **Responses, n (%)** | | | | |
| --- | --- | --- | --- | --- | --- |
|  | **Strongly agree** | **Agree** | **Neutral** | **Disagree** | **Strongly disagree** |
| It was easy to find a public toilet when I needed one | 52 (17.4%) | 107 (35.8%) | 68 (22.7%) | 54 (18.1%) | 18 (6.0%) |
| Most of the public toilets I used had a working flush | 33 (11.0%) | 87 (29.1%) | 77 (25.8%) | 84 (28.1%) | 18 (6.0%) |
| Most of the public toilets I used had a hand washing basin with soap and water or hand sanitiser | 22 (7.4%) | 79 (26.4%) | 67 (22.4%) | 87 (29.1%) | 44 (14.7%) |
| Most of the public toilets I used had hand drying facilities (paper towel or hand dryer) | 17 (5.7%) | 46 (15.4%) | 60 (20.1%) | 97 (32.4%) | 79 (26.4%) |
| My experiences of toilet and handwashing facilities were better than I expected | 21 (7.0%) | 63 (21.0%) | 130 (43.3%) | 57 (19.0%) | 29 (9.7%) |

**Table B: Frequency of travellers’ health symptoms**

| **Health Symptoms** | **Responses**  **n (%)** | | | |
| --- | --- | --- | --- | --- |
|  | **Always** | **Often** | **Sometimes** | **Rarely** |
| How often were you able to locate a facility to wash your hands with soap and water in the following situation (before eating)? | 134 (44.7%) | 113 (37.7%) | 39 (13.0%) | 14 (4.7%) |
| How often were you able to locate a facility to wash your hands with soap and water in the following situation (after going to the toilet)? | 145 (48.3%) | 95 (31.7%) | 45 (15.0%) | 15 (5.0%) |
| How often did you experience the symptom of 'nausea/vomiting' during your travel in India? | 3 (1.0%) | 17 (5.7%) | 67 (22.3%) | 213 (71.0%) |
| How often did you experience the symptom of 'loss of appetite' during your travel in India? | 2 (0.7%) | 24 (8.0%) | 70 (23.3%) | 204 (68.0%) |
| How often did you experience the symptom of 'diarrhoea/loose stools' during your travel in India? | 5 (3.6%) | 30 (21.9%) | 89 (65.0%) | 13 (9.5%) |
| How often did you experience the symptom of 'abdominal pain/cramping/stomach-ache' during your travel in India? | 5 (1.7%) | 31 (10.3%) | 72 (24.0%) | 192 (64.0%) |
| How often did you experience the symptom of 'bloody stool' during your travel in India? | - | 1 (0.3%) | 9 (3.0%) | 290 (96.7%) |
|  | **Yes** | **No** | **Maybe** | **Do not know** |
| Do you believe that these symptoms could be attributed to poor water, sanitation and hygiene that you experienced in India? | 120 (40.8%) | 95 (32.3%) | 68 (23.3%) | 11 (3.7%) |

| Gastro-intestinal symptoms | Receipt of medical advice prior to trip | | Odd’s Ratio  (95% CI) | Chi-square Value  P-value |
| --- | --- | --- | --- | --- |
|  | Yes | No |  |  |
| ^┼^**Occurrence of diarrhoea in last 7 days** |  | | | |
| No | 56 | 106 | Ref | 14.53  0.00^*^ |
| Yes | 78 | 60 | 0.4 (0.25-0.64) |  |
| **^┼^Loss of appetite** |  |  |  |  |
| Less frequent | 116 | 157 | Ref | 5.81  0.01^*^ |
| More frequent | 18 | 9 | 0.36(0.16-0.85) |  |
| **^┼^Abdominal pain** |  |  |  |  |
| Less frequent | 108 | 156 | Ref | 12.56  0.00^*^ |
| More frequent | 26 | 10 | 0.26(0.12-0.57) |  |
| **^┼^Nausea/Vomiting** |  |  |  |  |
| Less frequent | 121 | 159 | Ref | 3.58  0.006^*^ |
| More frequent | 13 | 7 | 0.41(0.15-1.08) |  |

Table C (I-IV): Distribution of travellers according to the association between gastro-intestinal symptoms and receipt of medical advice, type of accommodation, purpose of travel and duration of travel

Table C (I): Association between gastro-intestinal symptoms and receipt of medical advice prior to trip

*^┼^self-reported ^*^significance level <0.05*

Table C (II): Association between gastro-intestinal symptoms and type of accommodation

| Gastro-intestinal symptoms | Type of accommodation | | Odd’s Ratio  (95% CI) | Chi-square Value  P-value |
| --- | --- | --- | --- | --- |
|  | Hotel/Guesthouse | Other |  |  |
| ^┼^**Occurrence of diarrhoea in last 7 days** |  | | | |
| No | 110 | 52 | Ref | 7.725  0.005^*^ |
| Yes | 72 | 66 | 1.93 (1.21-3.10) |  |
| **^┼^Loss of appetite** |  |  |  |  |
| Less frequent | 164 | 109 | Ref | 0.448  0.503 |
| More frequent | 18 | 9 | 0.75(0.32-1.73) |  |
| **^┼^Abdominal pain** |  |  |  |  |
| Less frequent | 163 | 101 | Ref | 1.06  0.30 |
| More frequent | 19 | 17 | 1.44(0.71-2.9) |  |
| **^┼^Nausea/Vomiting** |  |  |  |  |
| Less frequent | 172 | 108 | Ref | 1.022  0.31 |
| More frequent | 10 | 10 | 1.59(0.64-3.95) |  |

*^┼^self-reported ^*^significance level <0.05*

Table C (III): Association between gastro-intestinal symptoms and purpose of travel

| Gastro-intestinal symptoms | Purpose of Travel | | Odd’s Ratio  (95% CI) | Chi-square Value  P-value |
| --- | --- | --- | --- | --- |
|  | Tourism | Other |  |  |
| ^┼^**Occurrence of diarrhoea in last 7 days** |  | | | |
| No | 105 | 57 | Ref | 3.88  0.04^*^ |
| Yes | 74 | 64 | 1.59(1.00-2.53) |  |
| **^┼^Loss of appetite** |  |  |  |  |
| Less frequent | 163 | 110 | Ref | 0.00  0.96 |
| More frequent | 16 | 11 | 1.01(0.45-2.27) |  |
| **^┼^Abdominal pain** |  |  |  |  |
| Less frequent | 160 | 104 | Ref | 0.80  0.3 |
| More frequent | 19 | 17 | 1.37(0.68-2.77) |  |
| **^┼^Nausea/Vomiting** |  |  |  |  |
| Less frequent | 168 | 112 | Ref | 0.194  0.66 |
| More frequent | 11 | 9 | 0.81(0.32-2.03) |  |

*^┼^self-reported ^*^ significance level <0.05*

Table C (IV): Association between gastro-intestinal symptoms and duration of travel

| Gastro-intestinal symptoms | Duration of Travel | | Odd’s Ratio  (95% CI) | Chi-square Value  P-value |
| --- | --- | --- | --- | --- |
|  | More than 2 weeks | Less than 2 weeks |  |  |
| ^┼^**Occurrence of diarrhoea in last 7 days** |  | | | |
| No | 113 | 49 | Ref | 7.56  0.006^*^ |
| Yes | 75 | 63 | 1.93(1.2-3.1) |  |
| **^┼^Abdominal Pain** |  |  |  |  |
| Less frequent | 165 | 99 | Ref | 0.26  0.87 |
| More frequent | 23 | 13 | 1.63(0.7-3.6) |  |
| **^┼^Loss of Appetite** |  |  |  |  |
| Less frequent | 174 | 99 | Ref | 1.44  0.22 |
| More frequent | 14 | 13 | 0.94(0.4-1.9) |  |
| **^┼^Nausea/Vomiting** |  |  |  |  |
| Less frequent | 178 | 102 | Ref | 1.47  0.23 |
| More frequent | 10 | 10 | 1.74(0.7-4.3) |  |

*^┼^self-reported ^*^significance level <0.05*
