# Supplementary material for: Effects of sanitation and hygiene perceptions on international travelers' health, travel plans and trip experiences in India
Source: Front Public Health. 2022 Nov 30;10:1042880. doi: 10.3389/fpubh.2022.1042880 (PMC9774491; doi:10.3389/fpubh.2022.1042880)
Supplement: Supplementary file 2 [file Table_2.DOCX]

**Appendix 1**

Dear participant,

Thank you for agreeing to take part in this survey **about you and your travel experiences in India**. Please **answer each question** by ticking the appropriate box. The survey should take about 10 minutes to complete.

*Eligibility: minimum stay 7 days in India*

*To be entered by research assistant*

1. *Questionnaire Code No : 2) Data Collection country & city:*

***Part A. Demographics & Travel characteristics***

1. Age

☐ less than 20 years ☐ 20-30 years ☐ 31-40 years ☐ 40 plus

1. Sex:

☐ Male ☐ Female ☐Other ☐ Prefer not to answer

1. Country of origin__________________.
2. Highest level of education

☐ Completed Primary School ☐ Completed Secondary School ☐ Completed University Degree

1. Ethnicity

☐ White ☐ Black ☐ Asian (Far East) ☐ Asian (Indian subcontinent) ☐ Hispanic/Latino ☐ Bi/Multi-racial ☐ Middle eastern

1. Purpose of travel

☐ Tourism/Vacation ☐ Business ☐ Educational/Research

☐ Volunteer/Missionary ☐ Visiting friends/Relatives ☐ other

1. Planned trip duration

☐ less than 1 week ☐ 1-2 weeks ☐ 3-4 weeks ☐ more than 4 weeks

1. Travelling partner(s) ☐ None/alone ☐ Friend(s)/family
2. Time already spent in India

☐ less than 2 weeks ☐ 2-4 weeks ☐ more than 4 weeks

1. Where are you staying currently?

☐ Hotel ☐ Guesthouse ☐ Home stay/ friend's home ☐Apartment ☐other

1. Do you have any underlying health condition?

☐ Yes ☐ No

1. Did you seek medical advice prior to your trip?

☐Yes, from my GP/family medicine doctor

☐Yes, from a specialised travel clinic

☐No

1. Have you ever received hepatitis A vaccination?

☐ Yes, within the last 12 months

☐ Yes, one dose more than 12 months ago

☐ Yes, two previous doses (completed course)

☐ No, never

1. Have you received typhoid vaccine within the past 3 years?

☐ Yes

☐ No

1. Which of the following medications are you carrying with you?

(please tick all that apply)

☐ Antimotility agent (loperamide) ☐ Oral rehydration solution/product

☐ Anti-emetic (nausea prevention) ☐ Probiotic

☐ Antibiotic ☐ Antiparasitic

***Part B. Water, Sanitation & Hygiene, and travel experiences***

1. What has been your preferred source of drinking water while travelling in India?

☐ Bottled water ☐ Boiled water ☐ Tap water

☐ Water treated with UV light ☐ Water treated with chlorine/iodine

1. Was your perception of sanitation experience in India ‘poor’ or ‘inadequate’?

☐ Yes ☐ No

1. Was your perception of hygiene experience in India ‘poor’ or ‘inadequate’?

☐ Yes ☐ No

1. Please indicate if you agree or disagree with each of the following statements about sanitation conditions in India (tick one box per statement only)

|  | Strongly agree | Agree | Neutral | Disagree | Strongly disagree |
| --- | --- | --- | --- | --- | --- |
| It was easy to find a public toilet when I needed one |  |  |  |  |  |
| Most of the public toilets I used had a working flush |  |  |  |  |  |
| Most of the public toilets I used had a hand washing basin with soap and water or hand sanitiser |  |  |  |  |  |
| Most of the public toilets I used had hand drying facilities (paper towel or hand dryer) |  |  |  |  |  |
| My experiences of toilet and handwashing facilities in India were better than I expected |  |  |  |  |  |

1. Please indicate how often you were able to locate a facility to wash your hands with soap and water in the following situations (tick one box for each activity)

|  | Always | Often | Sometimes | Rarely |
| --- | --- | --- | --- | --- |
| Before eating |  |  |  |  |
| After going to the toilet |  |  |  |  |

1. Did you experience of diarrhoea in the past week during your India trip?

☐ Yes ☐ No [if No, skip to question 23]

1. What was the severity of diarrhoea that you experienced?

☐ Mild (tolerable) ☐ Moderate (distressing) ☐ Strong (incapacitating)

1. Please indicate how often you experienced the following gastrointestinal illnesses during your travel in India (tick one box for each symptom, if applicable); if No, skip to question 30

|  | Always | Often | Sometimes | Rarely |
| --- | --- | --- | --- | --- |
| Nausea/vomiting |  |  |  |  |
| Loss of appetite |  |  |  |  |
| Diarrhoea/loose stools |  |  |  |  |
| Abdominal pain/cramping/stomachache |  |  |  |  |
| Bloody stool |  |  |  |  |

1. Do you believe that these symptoms could be attributed to poor water, sanitation and hygiene that you experienced in India?

☐ Yes ☐ No ☐ May be ☐ Do not know

1. Types of food you consumed before suffering such symptoms mentioned above

☐ Street food ☐ Food kept overnight ☐ Uncooked meat/fish

☐ Unwashed fruits/vegetables ☐ Bakery/sweets ☐ Other

1. What do you think was the main cause of your symptoms?

………………………………………………………………………………………………………………………………………………………………………………………………

1. Did these (your) symptoms affect your travel plans?

☐ Yes ☐ No

If No, skip to question 29

1. How were your travel plans affected by your symptoms?

☐ Stopped planned activities/ travelling ☐ Needed to change travel plans ☐ Needed medical consultation ☐ Needed hospitalizations

1. Did your symptoms adversely affect your overall travel experience?

☐ Yes ☐ No

1. Will you recommend anyone else to visit India considering the water, sanitation and hygiene facilities here?

☐ Yes ☐ No ☐ Not sure

1. What alteration in future travel plans due to perceptions of WASH experience in India would you consider?

☐Consideration of better accommodation

☐Avoiding going to countryside ☐ Other changes in itineraries………………………

1. Have your experiences of water, sanitation and hygiene in India influenced where you will choose to travel in the future?

☐ Yes ☐ No ☐ Not sure

Why/why not? Please elaborate…………………………………………………………………………………………………………………………………………………………………………………………………………………………………………………………………………………………………………………………………………………………………………………………..

1. Would you recommend India as a travel destination to your friends/family?

☐ Yes ☐ No ☐ Not sure

Why/why not? Please elaborate……………………………………………………………………………………………………………………………………………………………………………………………………………………………………………………………………………………………………………………………………………………………………………………………

**Thank you and wish you a nice travel in India ☺**
